# A Persian version of the Affiliate Stigma Scale in caregivers of people with dementia

**DOI:** 10.15171/hpp.2019.04

**Published:** 2019-01-23

**Authors:** Mohsen Saffari, Chung-Ying Lin, Harold G. Koenig, Keisha-Gaye N. O’Garo, Anders Broström, Amir H. Pakpour

**Affiliations:** ^1^Health Research Center, Life Style Institute, Baqiyatallah University of Medical Sciences, Tehran, Iran; ^2^Health Education Department, Faculty of Health, Baqiyatallah University of Medical Sciences, Tehran, Iran; ^3^Department of Rehabilitation Sciences, the Hong Kong Polytechnic University, Hung Hom, Hong Kong; ^4^Department of Psychiatry and Behavioral Sciences, Duke University Medical Center, Durham, NC, USA; ^5^Adjunct Professor, King Abdulaziz University, Jeddah, Saudi Arabia; ^6^Adjunct Professor, Ningxia Medical University, Yinchuan, China; ^7^Department of Nursing, School of Health and Welfare, Jönköping University, Jönköping, Sweden; ^8^Social Determinants of Health Research Center, Qazvin University of Medical Sciences, Qazvin, Iran

**Keywords:** Stigma, Family caregivers, Dementia, Psychometrics

## Abstract

**Background:** Dementia is prevalent among older adults and frequently causes dependence on family caregivers. Caregivers may experience a form of stigmatization called affiliate stigma that negatively affects their mental health. The current study sought to establish the psychometric properties of a tool to measure affiliate stigma among Iranian caregivers.

** Methods: ** Overall, 541 caregivers of older people with dementia were included in this cross sectional study. Several measures were used to assess the psychometric properties of the Affiliate Stigma Scale (ASS) including the Zarit Burden Interview (ZBI), Hospital Anxiety and Depression Scale (HADS), Short Form 12 (SF-12), Rosenberg Self-Esteem Scale (RSES), and Multidimensional Scale of Perceived Social Support (MSPSS). Convergent and discriminate validity were examined.Exploratory and confirmatory factor analyses were utilized to assess the factor structure of the Ass and a Rasch model was used to evaluate the measurement functioning of the scale.

**Results:** Factor loadings ranged from 0.69 to 0.83 and test-retest reliability from 0.72 to 0.89.Item difficulty ranged widely from -0.66 to 0.89. No considerable differential item functioning (DIF) was found across gender. Confirmatory factor analysis confirmed the three cognitive,effective, and behavioral dimensions of the scale (comparative fit index [CFI]=0.931 to 0.995,root mean square error of approximation [RMSEA]=0.046 to 0.068). Internal consistency was acceptable (Cronbach’s α: 0.88 to 0.94). Significant and positive relationships were found between affiliate stigma and depression, anxiety, and care giving burden (β =0.35 to 0.46).

** Conclusion: ** The ASS is a psychometrically valid measure for assessing affiliate stigma in Iranian caregivers of people with dementia. Application of this tool among other caregivers, language sand cultures deserves further study.

## Introduction

Dementia is a mental disorder that affects memory and cognition particularly among people age 65 years and older.^1^ More than 45 million people around the world suffer from dementia, and without an effective treatment, its prevalence may triple by 2050.^2^ This neurological disorder is present not only among older adults in developed areas of the world such as United States and Europe but it is also widespread in developing and underdeveloped regions of the world.^3^ Based on a recent report, nearly 8% of people over age 60 years old in Iran were diagnosed with dementia and in the Asia pacific region more generally, nearly 23 million people had dementia in 2015.^4,5^

Although people with dementia may have many emotional, cognitive, social, and physical health problems, their caregivers also our affected by issues related to caregiving such as stress, reduced quality of life, depression, anxiety, deprivation of a normal lifestyle, and inadequate leisure time that may cause considerable caregiver burden.^6^ This burden may worsen when the cared for person has inappropriate or bizarre symptoms unfamiliar to the caregiver and others who may come into contact with the person.^7^ Caregivers, then, become susceptible to several types of stigmatization.^8^

Stigmatization is a process in which a person or group unjustifiably shames, excludes or discriminates against another person. Stigma has been defined as an attribution that makes an individual different from others who are in a similar category and therefore marks them as unusual.^9^ There are several kinds of stigma including “bodily disgrace stigma,” caused by losing a body limb; “moral incapability stigma,” where abnormal thoughts or behaviors make the person different than those around him/her; or “courtesy stigma” that occurs when the individual is affiliated with a stigmatized person or group.^10^ The last type (i.e., courtesy stigma) may be divided into two subcategories: public stigma and affiliate stigma. Public stigma indicates negative reactions from people in society toward stigmatized people, and when these negative reactions are internalized by the people exposed to stigma, it is called affiliate stigma.^11^

Affiliate stigma in the case of dementia caregivers is the product of a self-stigmatization process by which caregivers, because of their association with people who have dementia, are exposed to social prejudice, stereotypes and discrimination.^12^ When stigmatized caregivers are family members of people with dementia, a specific type of affiliate stigma may be experienced called family stigma.^11^ Unfortunately, this type of stigmatization increases social withdrawal by caregivers and contributes to the development of psychological disorders such as depression, anxiety and poor self-esteem.^7,8^ Despite the negative impact of this stigma on caregiver health status, there is only limited research on this topic, largely due to a lack of valid and reliable instruments to assess this form of stigmatization.^12^

To our knowledge, only two studies have thus far sought to develop measures for identifying affiliate stigma among caregivers of people with dementia. Family Stigma in Alzheimer’s Disease (FSAD) is a 42-item scale that assesses affiliate stigma among caregivers who are children of the people with Alzheimer’s disease.^13^ Another tool is the Affiliate Stigma Scale (ASS), which is a 22-item measure with a wider target group – all dementia caregivers, not just caregivers of those with Alzheimer’s disease. This theory driven instrument was designed for easier use in clinical settings due to fewer items, and is appropriate for a wide range of family caregivers including children, spouses, grandchildren, and other relatives.^11,12^ The ASS measure also has other advantages including well-established validity and utility for caregivers of those with other psychiatric disorders such as schizophrenia and people with mood disorders.^14^

In the Iranian culture where the majority of people are Muslim, similar to many other cultures and religions, respect for elders – especially parents or close relatives – is highly valued. In addition, based on Islamic teachings that stress the concept of altruism, when dealing with people who are disadvantaged through some disability, others should make their best efforts to help or support the disadvantaged person.^15^ Therefore, because there are no Persian measures to assess affiliate stigma among Iranian caregivers of people with dementia, we felt that the ASS might be ideal for capturing this form of stigma among Iranian caregivers. Consequently, we decided to culturally adapt this scale to the Iranian context.

## Materials and Methods

### Design, participants, and procedure

This cross-sectional study was performed during a six-month period from December 2017 to May 2018. Participants were 541 caregivers of patients with dementia who were registered in the Iran Alzheimer’s Association (IAA) to receive routine care in Tehran and Qazvin. Sample size was calculated based on 20 or more subjects per item. All accessible and qualified persons were invited to participate (convenience sampling). Inclusion criteria for the care recipients were (1) age 65 or over and (2) confirmed diagnosis of dementia according to ICD-criteria. Inclusion criteria for the caregivers were (1) age of 18 years or over, (2) primary caregiver of a family member with dementia, and (3) completion of informed consent for participation. Caregivers with cognitive impairment (assessed by Mini-Mental State Examination) or any mental disability and non-family caregivers were excluded. Inclusion and exclusion criteria were assessed by two physicians during a screening evaluation. The questionnaire took about 20-30 minutes to complete. We explained that the purpose of the study was to develop a scale to identify and resolve problems related to affiliate stigma in caregivers, and encouraged participants to answer all questions to the best of their ability. Participation was completely voluntarily, and participants were told that they could stop at any time. Questionnaires were completed at the time of a clinic visit in the clinic.

### Translation 

International guidelines were followed when translating the English version of the ASS into Persian.^16^ In the first step, two forward-translations (from English to Persian language) were performed separately by two bilingual translators whose mother tongue was Persian. The two translators and a recording observer compared the translated with the original questionnaire and arrived by consensus at a preliminary Persian version. This version was then translated back into English by two persons with English as their original language. Translators acted independently and were not aware of the original English version. All translations were then reviewed by an expert committee including a psychologist, psychometrician, nurse, neurologist, and gerontologist together with the translators. The committee arrived at a consensus version after checking the similarity of each item between English and Persian versions. This consensus version was then piloted in 33 caregivers of patients with dementia (mean age = 56.3, 63.6% female) to explore the meaning of the items and responses from their standpoint. These participants were excluded from the main study. After making minor changes, the final version was arrived at and administered to 541 caregivers of patients with dementia.

### Measures

Affiliate Stigma Scale (ASS). The ASS contains 22 items across three domains (cognitive with 7 items; affective with 7 items; and behavioral with 8 items), with response options ranging from 1 to 4 on a Likert scale. Higher scores on the ASS indicate greater caregiver self-stigma. Previous studies have demonstrated satisfactory psychometric properties for the ASS in a wide range of different populations, including caregivers of people with dementia.^11,12,14^

Zarit Burden Interview (ZBI). The ZBI contains 22 items rated on a 4-point Likert scale (1-4). The ZBI has been found to have different factor structures when measuring caregiver burden^17^; however, most indicate a single factor.^18^ A higher score on the ZBI indicates greater caregiver perceived burden. The Persian version of the ZBI has been shown to have acceptable psychometric properties (e.g., Cronbach’s alpha = 0.78).^19^

Hospital Anxiety and Depression Scale (HADS). The HADS contains 14 items across two subscales measuring anxiety and depression with 7 items each, with each item having a 4-point Likert scale (0-3) response option. A higher score on the HADS indicates greater anxiety or depression. The Persian version of the HADS has been shown to have solid psychometric properties. ^20^

Short Form 12 (SF-12). The SF-12 contains 12 items separated across two domains assessing quality of life, and produces physical health and mental-health composite scores (PCS and MCS), each assessed by 6 items. The SF-12 scoring algorithm produces composite scores with a mean score of 50.0 and a SD of 10.0 using global norms, with a range from 0 to 100, where higher scores indicate better quality of life for either PCS or MCS. The Persian version of the SF-12 also has good psychometric properties and has been shown to distinguish participants based on gender and education. The original factor structure of the English SF-12 has been confirmed for the Persian SF-12.^21^

Rosenberg Self-Esteem Scale (RSES). The RSES contains 10 items producing a single factor, with response options on a 4-point Likert scale (1-4). Higher scores indicate better self-esteem. The Persian version of the RSES has been shown to have solid psychometric properties indicating a single factor.^22^

Multidimensional Scale of Perceived Social Support (MSPSS). The MSPSS contains 12 items across three subscales (family, friends, and significant others, with 4 items for each subscale), with response options on a 7-point Likert scale (1-7). Higher scores indicate greater perceptions of social support from a specific resource (i.e., family, friends, or significant others). The Persian version of the MSPSS has adequate psychometric properties (e.g., Cronbach’s alpha ranging from 0.87 to 0.92).^23^

### Data analysis

Mean, SD, and frequency distributions describe the demographic characteristics of participants. Ceiling (the proportion of participants who achieved the maximum score) and floor (the proportion of participants who achieved the minimum score) effects were computed to assess measurement range, with values of 15% or more indicating the presence of ceiling or floor effects.

Reliability of the ASS was determined by internal consistency and test retest reliability over a 2-week interval, using Cronbach’s alpha and intraclass correlation coefficients (ICC), respectively. A value of 0.70 or higher for both Cronbach’s alpha and ICC is considered acceptable reliability. Factor structure of the ASS was assessed using confirmatory factor analysis (CFA) for each domain and for the entire scale. Weighted least squares means and variance adjusted (WLSMV) estimator was used to determine ordinal indicators of the data. Several model fit indices were used to evaluate the appropriateness of the factor structure, including chi square index χ^2^, the root mean square error of approximation (RMSEA), Tucker-Lewis index (TLI), the comparative fit index (CFI), the standardized root mean square residual (SRMR), and the weighted root-mean-square residual (WRMR). Acceptable cutoffs for model fit are a non-significant χ^2^ (*P *> 0.05), RMSEA <0.08, CFI and TLI >0.90 and WRMR <1.0. The average variance extracted (AVE) and composite reliability (CR) were also calculated to assess the convergent validity for each latent variable. An AVE ≥ 0.50 and a CR > 0.6 are considered acceptable convergent validity.

Rasch partial credit model was performed to obtain item difficulty estimates, including item validity, item and person separation reliabilities, and item and person separation indices. The Rasch analysis converts the ASS raw item scores into interval logit measures, with higher logit values indicating more item difficulty. Item validity was measured using information-weighted fit statistic (infit) mean square (MnSq) and outlier-sensitive fit statistic (outfit) MnSq, with values between 0.5-1.5 indicating acceptable fit for the Rasch Model (values outside of this range indicate either redundancy or misfit, <0.5 and >1.5, respectively). Item and person reliabilities were also obtained using the Rasch analysis, with values greater than 0.70 indicating acceptable reliability. Item and person separation indices were also estimated to determine item validity, with values greater than 2 considered acceptable.

In order to ensure that the probability of response to an item was equal across genders, multigroup CFA and differential item functioning (DIF) were performed.

The 3-factor structure of the Affiliated stigma scale was first evaluated by running multigroup CFA across male and female caregivers using three nested models: configural invariance model (all items loading on their corresponding latent concept), metric invariance model (a model constraining all factor loadings to be equal across genders), and a scalar invariance model (based on the metric invariance model constraining all item intercepts to be equal across genders). Measurement invariance is supported when ΔCFI values ≤0.1, ΔRMSEA<0.015, and ΔSRMR<0.01 in model comparisons.

Measurement invariance was further tested with DIF across genders, where DIF of <0.5, 0.5–1.0, and >1.0 logits are considered as inconsequential, minimal, and significant, respectively.

The validity of the ASS was further assessed using corrected item-total correlation and criterion-related validity. A value of >0.4 is considered acceptable for determining whether each item is well connected to the entire concept. Criterion-related validity was assessed using multiple linear regression with ASS total score as the dependent variable and anxiety, depression, perceived social support, self-esteem, caregiver burden, and health-related quality of life as independent variables, controlling for age, gender and duration of caregiving. Standardized regression coefficient (β) was used to assess associations between ASS total score as the outcome and other measures as predictors. All statistical analyses were performed using MPLUS 7.2 software and Winstep version 3.91.0.

## Results

A total of 541 patient-caregiver dyads were recruited (demographic information presented in Table 1). For caregivers, their mean (SD) age was 59.4 (12.0) and average years of education was 7.2 (5.1). Approximately one third of caregivers were male (32.9%) and one-half were married (48.1%). Most caregivers were children (61.2%) or spouses (32.0%) of the people with dementia. Average duration of caregiving was 48.6 (15.9) months, and average hours per week spent in caregiving was 64.3 (18.7) hours. Average scores on burden, anxiety, depression, self-esteem, perceived social support, and quality of life are displayed in Table 1. Regarding patients (care recipients), mean age was 71.4 (15.7), and average years of education was 3.1 (0.9). Approximately two-thirds were male (61.0%) and more than half were currently married (55.8%). Table 1 also presents average cognitive functioning measured using the MMSE and physical functioning based on instrumental activity of daily living.

Psychometric properties of the ASS at the item level are presented in Table 2. All items had strong factor loadings (0.69 to 0.83), high item-to-total correlation (0.57 to 0.81), satisfactory test-retest reliability (0.72 to 0.89), and adequate fit indices (infit MnSq = 0.77 to 1.31; outfit MnSq = 0.75 to 1.34). Item difficulty had a relatively wide range from -0.66 to 0.89. All but three items (#1 in cognitive domain; #1 and #2 in the affective domain) did not show substantial DIF across genders.

Psychometric properties of the ASS at the scale level are presented in Tables 3 through 6. Table 3 indicates that the three domains of the ASS (i.e., cognitive, affective, and behavioral) and the entire ASS had adequate model fit indices (CFI = 0.931 to 0.995; TLI = 0.924 to 0.990; RMSEA = 0.046 to 0.068; WRMR = 0.75 to 0.88; SRMR = 0.021 to 0.055) for CFA, except for the significant χ^2^ tests. Table 4 provides additional psychometric properties for the ASS at the scale level: high CR (0.90 to 0.97) and AVE (0.53 to 0.62); low ceiling (5.7% to 6.8%) and floor effects (5.2% to 7.2%); excellent Cronbach’s α (0.88 to 0.94); satisfactory separation reliability (person separation reliability = 0.82 to 0.93; item separation reliability = 0.89 to 1.00); acceptable separation indices (person separate index = 2.14 to 3.62; item separation index = 2.78 to 17.61); and standard error of measurements (SEMs) that were lower than half the SDs. Criterion-related validity of the ASS was demonstrated based on moderate correlations with depression, anxiety, quality of life, caregiving burden, self-esteem, and perceived social support (Table 5). Controlling analyses for age and gender (including these variables in a separate block in the regression model), depression (β = 0.35), anxiety (β = 0.46), and caregiving burden (β = 0.35) were significantly and positively associated with the ASS total score, whereas quality of life (β = -0.35 for PCS and -0.33 for MCS), self-esteem (β = -0.23), and perceived social support (β = -0.60) were significantly and inversely associated with ASS total score.

Since the 3-factor structure of the ASS was supported by CFA, examination of measurement invariance was performed to check whether male and female caregiver scores on the ASS were similar in structure. The metric invariance model was not significantly different from the configural invariance model (∆χ^2^ = 30.404; ∆df = 22; *P* = 0.11), and scalar invariance model was not significantly different from the metric invariance model (∆χ^2^ = 27.192; ∆df = 22; *P* = 0.201). Other fit indices also demonstrated measurement invariance for the ASS across gender (Table 6).

## Discussion

The aim of the current study was to determine the psychometric properties of a culturally and linguistically adapted version of a measure of affiliate stigma among Iranian caregivers of patients with dementia. We found that the ASS is an appropriate tool for assessing this construct among caregivers in Iran. The original factor structure of the scale that identified cognitive, affective, and behavioral domains was replicated. The scale also demonstrated internal consistency and both convergent and divergent validity. Discriminate validity of the scale was demonstrated by small to moderate correlations with measures of depression, anxiety, quality of life, social support and self-esteem. The factor analysis and invariance analysis indicated that the scale may be useful in both genders. There was also a wide range in terms of difficulty level, allowing for use among caregivers from a broad range of education levels.

The significant number of dementia caregivers in our sample provides important characteristics of such individuals that may be compared to other studies. For example, in many studies conducted on this population (as we found here), female caregivers were more prevalent than male caregivers.^7,8,24^ This makes sense given that the tendency for women to be more companionate and nurturing, especially towards other family members. However, in Iranian culture and the Muslim religion (as in many other faith traditions), women are expected to serve as caregivers because one of their most important duties is providing care for other members of the family especially when they have health problems. The average age of patients in our sample was 70 years, which is comparable with the average age that dementia often begins among older adults in Iran, when caregiving begins.^4^ This is similar to other studies of people with dementia and their caregivers conducted in other regions of the world and cultures.^6^

As indicated by the original developers of the ASS, this instrument is based on cognitive and behavioral theory.^11^ The strong factor loadings and fit indices of the proposed model was replicated by our confirmatory factor analysis and supports consistency of the scale with its conceptual model, indicating that the ASS is a theory-driven tool that accurately and reliable assesses affiliate stigma as hypothesized. In addition, because previous research on ASS focused on different caregiver populations of people with intellectual disabilities and mental illnesses^12^ (as well as dementia in this study), this indicates that the scale may be used for caregivers of persons with a wide range of different health issues where stigmatization of caregivers is possible.

We also assessed associations between affiliate stigma and mental health indicators such as quality of life, self-esteem, caregiver burden, depression, and anxiety. Chang et al also examined these associations among caregivers of patients with various levels of severity of mental illness in Taiwan. They found similar associations as reported in the current study, noting that affiliate stigma among caregivers of patients with schizophrenia is greater than that of caregivers of patients with bipolar disorder or depression.^25^ Therefore, this scale may differentiate between caregivers of those with different severity of illness demonstrating known-group validity of the scale.

A new aspect of the scale not previously reported was the wide range of item difficulty. This suggests the ASS may be a practical scale for use among people with different levels of education and is not specific only for those with either low or high education. This feature may contribute to the comprehensiveness of the ASS for assessing affiliate stigma among caregivers with a wide range of knowledge levels. Another new finding was the acceptable DIF across genders. In other words, most items on the ASS are not biased toward one gender or the other. This means that the scale may be used in caregivers regardless of gender and increases the comparability of the scale in caregiver populations with different proportions of males and females.

We found a significant negative correlation between social support and affiliate stigma, which is of considerable importance given that social support is an important factor in enabling caregivers to continue with their caregiving duties. Ma and Mac examined caregivers of children with physical disabilities and also found a significant association between these two parameters.^26^ This suggests that social support may be a key factor in helping those with affiliate stigma, both for our group of caregivers of those with dementia and for those of young children. Thus, the ASS may be a useful tool for assessing affiliate stigma and caregivers of patients of different ages, which should be further explored in future studies conducted in other religions and cultures.

The present study also had a number of limitations that should be acknowledged because these may influence the design of future studies on this topic. First, we limited our sample to caregivers referred by the IAA. Our findings, then, may not be generalizable to all Iranian caregivers of people with dementia. Affiliate stigma may be different between caregivers who receive support from IAA (that majority being well-off Iranian citizens) and those who live in rural deprived areas. Second, as several studies have emphasized, dementia may occur among individuals younger than age 65 and the etiology of those dementias may be different than for those that develop among those over age 65, and behavioral issues may vary as well.^27,28^ Given our focus on caregivers of older persons with dementia, the results may differ among caregivers of those who are middle-aged or younger with dementia. Third, other factors such as illness severity or specific types of dementia not measured in this study may affect affiliate stigma and therefore should be assessed in future studies examining the psychometric characteristics of the ASS. Finally, criterion-related validity of the ASS could be assessed more completely by administering other validated scales of this construct (affiliate stigma). Nevertheless, since no validated scale of this type in Persian currently exists, it was necessary compare the scale with non-specific measures such as quality of life and caregiving burden.

## Conclusion

The Persian ASS is a valid and reliable measure for use in Iranian caregivers of people with dementia. We found significant associations between the ASS scale and other caregiver characteristics such as quality of life, depression, anxiety, self-esteem, and social support, suggesting that either affiliate stigma may affect these different health characteristics or that these health characteristics may affect this type of stigmatization. If the former is found to be true in future studies, mental health professionals may need to focus on reducing this form of stigma among caregivers in their attempts to improve the mental health of this population. Research on use of the ASS among caregivers of people with other mental, neurological, or physical health problems and those who live in the different cultures and religious environments, particularly longitudinal studies, should be a priority.

## Ethical approval

The protocol of the study was approved by the institutional review board of Qazvin University of Medical Sciences (ID: REC.12/17359). In addition, participants were informed of the research procedure, comprehensive information on the objectives; they also were informed about confidentiality, privacy, the right to end their participation and benefits. A signed informed consent form was obtained from all participants before data collection. All questionnaires were anonymous, and files that included participants’ contact were shredded after all data were collected. Only the research-related personnel could access and use the data. The study was conducted according to the World Medical Association Declaration of Helsinki.

## Competing interests

The authors declare that they have no competing interests.

## Authors’ contributions

All authors read and approved the final manuscript. MS, AHP and C-Y L conceived of the study and participated in the design and data collection. AB, HGK and K-G N O participated in the data analyses and MS preparation.

## Acknowledgments

The authors would like to express our thanks to the administration and staff of Iran Alzheimer Association for their kind cooperation with the research team in successfully completing this study.

**Table 1:** Participant characteristics (n = 541)

**Characteristics**	**Caregiver**	**Care recipient**
	**No. (%) or M (SD)**	**No. (%) or M (SD)**
Age (y)	59.35 (12.03)	71.36 (15.72)
Gender (male)	178 (32.90%)	330 (61.0%)
Years of education	7.23 (5.08)	3.12 (0.86)
Marital status		
Single	167 (30.87%)	10 (1.85%)
Married	260 (48.06%)	302 (55.82%)
Widowed	114 (21.07%)	229 (42.33%)
Accommodation		
Rural	92 (17.0 %)	124 (22.92%)
Urban	449 (83.0%)	417 (77.08%)
Relationship to care recipient		
Spouse	173 (31.98%)	-
Child	331 (61.18 %)	-
Others	37 (6.84 %)	-
Occupation		
Employed	238 (43.99 %)	-
Retired	97 (17.93%)	416 (76.89%)
Never been employed or others	206 (38.08%)	125 (23.10%)
No. of comorbidities^c^		
None	373 (68.94%)	-
One	86 (15.90%)	114 (21.07 %)
Two	54 (9.98%)	351 (64.88%)
Three or more	28 (5.18 %)	76 (14.05%)
Duration of caregiving (months)	48.62(15.93)	-
Hours per week of care	64.32(18.73)	-
Mini mental state examination	-	17.60(5.26)
ZBI	29.31(10.89)	-
Anxiety^a^	8.73 (4.45)	-
Depression^a^	7.12 (5.01)	-
RSES	20.14(3.21)	
MSPSS	58.31(11.76)	
Physical-health composite summary^b^	63.01(7.12)	-
Mental-health composite summary^b^	50.92(6.18)	-

Abbreviations: MSPSS, Multidimensional Scale of Perceived Social Support; ZBI, Zarit Burden Interview; RSES, Rosenberg Self-Esteem Scale. ^a^ Measured using Hospital Anxiety and Depression Scale. ^b^ Measured using Short-Form 12. ^c^Hypertension, coronary disease, diabetes mellitus, etc.

**Table 2:** Psychometric properties of the Affiliate Stigma Scale at the item level

**Item No.**	**Analyses from classical test theory**			**Analyses from Rasch**			
	**Factor loading** ^a^	**Item-total correlation**	**Test-retest reliability** ^b^	**Infit MnSq**	**Outfit MnSq**	**Difficulty**	**DIF contrast across gender** ^cd^
Congnitive-1	0.71	0.64	0.72	1.10	1.02	0.09	0.01
Congnitive-2	0.82	0.74	0.78	0.83	0.76	-0.12	**-0.59**
Congnitive-3	0.77	0.70	0.83	0.90	0.83	0.32	0.29
Congnitive-4	0.80	0.73	0.77	0.87	0.85	0.01	0.07
Congnitive-5	0.70	0.68	0.79	1.02	1.01	0.13	0.03
Congnitive-6	0.69	0.65	0.84	1.31	1.31	-0.07	0.01
Congnitive-7	0.77	0.73	0.88	0.96	0.88	-0.36	-0.27
Affective-1	0.79	0.76	0.79	0.86	0.85	-0.64	**-0.55**
Affective-2	0.82	0.79	0.74	1.06	1.04	-0.39	**-0.61**
Affective-3	0.74	0.71	0.76	1.01	1.01	0.48	0.43
Affective-4	0.80	0.77	0.86	0.77	0.80	0.29	-0.29
Affective-5	0.81	0.77	0.80	1.28	1.34	0.49	0.38
Affective-6	0.83	0.78	0.83	0.89	0.89	0.06	0.49
Affective-7	0.71	0.70	0.89	1.02	1.03	-0.30	0.39
Behavioral-1	0.70	0.57	0.82	0.86	0.93	-0.66	0.08
Behavioral-2	0.72	0.81	0.79	1.17	1.13	0.24	0.19
Behavioral-3	0.78	0.72	0.76	0.84	0.75	-0.08	-0.25
Behavioral-4	0.77	0.62	0.84	0.86	0.84	-0.19	-0.38
Behavioral-5	0.72	0.60	0.72	0.84	0.85	0.20	-0.09
Behavioral-6	0.69	0.74	0.77	1.06	1.06	0.89	-0.47
Behavioral-7	0.71	0.62	0.76	1.13	1.03	-0.40	-0.11
Behavioral-8	0.72	0.76	0.88	1.15	1.13	0.01	-0.47

Abbreviation: DIF, differential item functioning. ^a^ Based on the Second-order confirmatory factor analysis. ^b^ Using Intraclass Correlation Coefficient (ICC). ^c^ DIF contrast > 0.5 indicates substantial DIF. ^d^ DIF contrast across gender=Difficulty for females-Difficulty for males.

**Table 3:** Goodness-of-fit indices for confirmatory factor analysis of the Affiliate Stigma Scale

**Psychometric testing**	**Cognitive**	**Affective**	**Behavioral**	**Entire scale **
χ^2^(*df*)	30.07 (14)*	23.42 (14)*	70.05 (20)*	676.49 (206)*
CFI	0.962	0.995	0.952	0.931
TLI	0.944	0.990	0.933	0.924
RMSEA	0.054	0.046	0.068	0.065
Weighted root mean square residual	0.84	0.75	0.88	0.85
SRMR	0.046	0.021	0.055	0.048

Abbreviations: CFI, Comparative Fit Index; RMSEA, root mean square error of approximation; SRMR, Standardized Root Mean Square Residual; TLI, Tucker-Lewis index. *P < 0.05.

**Table 4:** Psychometric properties of the Affiliate Stigma Scale at the domain level

**Psychometric testing**	**Cognitive**	**Affective**	**Behavioral**	**Entire scale**
CR	0.90	0.92	0.90	0.97
AVE	0.57	0.62	0.53	0.57
Ceiling effects (%)	6.83	9.92	5.73	6.81
Floor effects (%)	7.21	5.17	7.39	5.92
Internal consistency (Cronbach’s α)	0.89	0.92	0.88	0.94
Person separation reliability	0.82	0.87	0.87	0.93
Person separation index	2.14	2.60	2.63	3.62
Item separation reliability	0.89	0.98	0.97	1.00
Item separation index	2.78	6.48	5.77	17.61
Standard error of measurement (SEM)	0.27	0.28	0.23	0.23
Mean (SD)	2.57 (0.82)	3.49 (0.99)	4.06 (0.65)	3.40 (0.70)

Abbreviations: CR, composite reliability; AVE, average variance extracted.

**Table 5:** Criterion-related validity of the Affiliate Stigma Scale using regression models with adjustments for age and gender

**Criterion**	**B (SE)**	**β**	**95% CI**	
			Lower	Upper
Depression^a^	0.40 (0.01)	0.35	0.38	0.42
Anxiety^a^	0.32 (0.01)	0.46	0.30	0.34
Physical-health Composite Summary^b^	‏-0.27 (0.02)	‏-0.35	-0.30	‏-0.25
Mental-health Composite Summary^b^	-0.23 (0.01)	-0.33	-0.28	0.23
ZBI	0.27 (0.02)	0.35	0.25	0.30
RSES	-0.20(0.03)	-0.40	-0.23	-0.17
MSPSS	-0.34 (0.02)	-0.60	-0.38	-0.31

Abbreviations: CI, confidence interval; ZBI, Zarit Burden Interview; MSPSS, Multidimensional Scale of Perceived Social Support; RSES, Rosenberg Self-Esteem Scale. ^a^Depression and anxiety were measured using Hospital Anxiety and Depression Scale. ^b^ Physical- and mental-health composite summaries were measured using Short Form 12.

**Table 6:** Measurement invariance across gender and living condition on Affiliate Stigma Scale using confirmatory factor analysis

**Model and comparisons**	**Fit statistics**							
	**χ** ^ 2 ^ ** (** ***df*** **)**	**∆χ** ^ 2 ^ **(∆df)**	**CFI**	**∆CFI**	**SRMR**	**∆SRMR**	**RMSEA**	**∆RMSEA**
Gender								
M1: Configural	1061.538(412)*		0.963		0.057		0.054	
M2: Plus all loadings constrained	1091.942(434)*		0.960		0.061		0.058	
M3: Plus all intercepts constrained	1329.134 (456)*		0.956		0.064		0.060	
M2−M1		30.404 (22)		-0.003		0.004		0.004
M3−M2		27.192 (22)		-0.004		0.003		0.002

Abbreviations: CFI, comparative fit index; SRMR, standardized root mean square residual; RMSEA, root mean square error of approximation. M1 = Model 1, a configural model; M2 = Model 2, a model based on M1 with all factor loadings constrained being equal across groups; M3 = Model 3, a model based on M2 with all item intercepts constrained being equal across groups. *P < 0.05
